# Microsporidial Keratoconjunctivitis Outbreak among Athletes from Hong Kong Who Visited Singapore, 2012

**DOI:** 10.3201/eid1903.121150

**Published:** 2013-03

**Authors:** Tsz-sum Lam, Man-ha Wong, Shuk-kwan Chuang

**Affiliations:** Author affiliation: Department of Health, Hong Kong Special Administrative Region, People’s Republic of China

**Keywords:** Microsporidia, Vittaforma species, keratoconjunctivitis, parasitic protists, parasite, fungi, Hong Kong, People’s Republic of China, Singapore, Malaysia, Australia, United Arab Emirates, rugby, athlete, tournament, soil, dirt, mud

**To the Editor:** An international outbreak of microsporidial keratoconjunctivitis related to soil contact in a Singapore sport venue during April was reported to Hong Kong Department of Health, People’s Republic of China, in May 2012. Microsporidia are obligate intracellular, unicellular, eukaryotic, parasitic protists related to fungi (*1*). Fourteen species of microsporidia have been detected in humans (*1*). Several species of microsporidia, such as *Vittaforma corneae*, can cause keratoconjunctivitis (*1*). An increasing incidence of microsporidial keratitis in Singapore that is strongly correlated with exposure to soil was reported during 2004–2007 (*2*). In a case series of 22 patients during 2006–2008 in Singapore, soil or mud were reported as predominant ocular contaminants that were contacted by athletes during sporting activities, such as playing rugby in muddy fields (*3*).

The outbreak reported in May 2012 affected 34 (41%) of 82 rugby players from Hong Kong who had participated in a rugby tournament in Singapore during April 21–22, 2012. In addition to the affected athletes from Hong Kong, there were 89, 15, 13, and 9 affected players, respectively, from Singapore, Malaysia, Australia, and United Arab Emirates (*4*). We conducted a retrospective cohort study among players from Hong Kong to identify potential risk and preventive factors for microsporidial keratoconjunctivitis.

The rugby tournament involved ≈1,600 boys and girls from 16 rugby clubs in Singapore, Hong Kong, Malaysia, Australia, and the United Arab Emirates. We invited 82 boys (8–16 years of age) from 2 Hong Kong rugby clubs that participated in the tournament to participate in telephone interviews during May 18–25, 2012. Using a standardized questionnaire, we collected information describing demographics and potential risk and preventive factors. We defined a case-patient as any player who had eye redness and 1 of the following ocular signs or symptoms since April 21: pain, discharge, swelling, or itchiness. 

We interviewed 73 (89%) of the 82 players: 34 (47%) met the case definition. The median age of case-patients was 13 years (range 9–16 years); these figures were not different from those of the cohort (median age 13 years, range 8–16 years).

Onset of the reported 34 cases ranged from April 26 through May 22, peaking on May 7 (Figure). The distribution of onset of cases over time indicates a point-source outbreak and reflects a wide range of incubation periods. Symptom onset occurred a median of 15 days (range 5–31 days) after opening day of the tournament.

**Figure:**
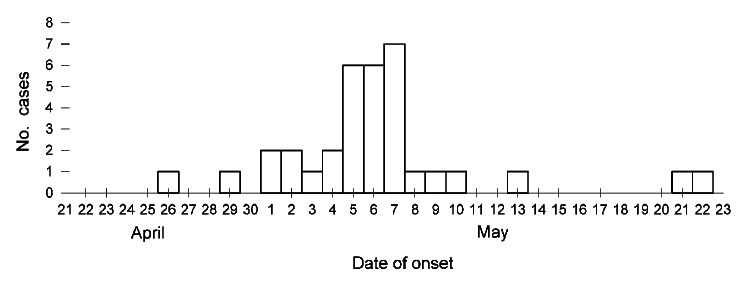
Onset dates of 34 cases of microsporidial keratoconjunctivitis among rugby players from Hong Kong, People’s Republic of China, who were exposed to contaminated soil and mud during a tournament in Singapore, April 21–22, 2012. Three cases (onset May 4, 5, and 7) were diagnosed in players by positive PCR testing; all other cases were diagnosed by the presence of eye redness and 1 of the following ocular signs or symptoms since April 21: pain, discharge, swelling, or itchiness.

Except for 2 players who had histories of asthma, all players reported good past health. Ocular signs and symptoms of the 34 case-patients were redness (100%), pain (53%), itchiness (53%), discharge (47%), and swelling (41%). Corneal scraping samples from 3 players were positive for *V. corneae* by PCR.

Heavy rainfall on playing fields was reported on April 21, when all the players from Hong Kong participated in the games and were exposed to soil and muddy water. Some players washed their eyes after exposure to the dirt in the field. We identified the following as preventive factors for keratoconjunctivitis: individual eye washing by bottled or tap water (relative risk [RR] 0.38, 95% CI 0.23–0.62), bottled water (RR 0.44, 95% CI 0.25–0.76) and tap water (RR 0.50, 95% CI 0.27–0.92). Group eye washing with water from a hose held by a trainer was not preventive. It is possible that individual eye washing by the players was more thorough.

The 47% attack rate among the players from Hong Kong is higher than the overall 10% (160/1,600) attack rate for tournament participants. Rugby players from Hong Kong might have been less aware of the risk of contracting microsporidial keratoconjunctivitis through soil or muddy water exposure than were players from other locations.

This outbreak provided an opportunity to study the incubation period of *V. corneae* keratoconjunctivitis in otherwise healthy persons. Time from soil exposure to development of ocular symptoms of microsporidial keratitis has been reported to be 2–21 days (median 14 days) (*2*) and 5–14 days (mean 6.8 days) (*3*). The incubation period during this outbreak was 5–31 days (median 15 days).

An investigation by the Singapore Ministry of Health of this outbreak revealed that microsporidial spores are probably ubiquitous in soil in Singapore (*4*). All interviewed rugby players from Hong Kong were exposed to soil or muddy water in Singapore before the outbreak, but we could not demonstrate the dose–response relationship because of the long incubation period and difficulty quantifying exposure to soil and muddy water.

This was an uncommon microsporidial keratoconjunctivitis outbreak. Advice about hygiene should be given to athletes who are exposed to dirt and mud on playing fields to minimize their risk for infection. Instructions should be given for safe and thorough washing of eyes, especially after dirt/mud exposures on waterlogged playing fields. Clinicians and public health professionals must consider microsporidial keratoconjunctivitis as a differential diagnosis for conditions of field athletes who exhibit eye redness accompanied by eye pain, discharge, swelling, or itchiness after exposure to soil or mud.
